# GRP78 Protein Expression in Ovarian Cancer Patients and Perspectives for a Drug-Targeting Approach

**DOI:** 10.1155/2012/468615

**Published:** 2012-03-18

**Authors:** Florence Delie, Patrick Petignat, Marie Cohen

**Affiliations:** ^1^School of Pharmaceutical Sciences, University of Geneva/university of Lausanne, Quai Ernest Ansermet 30, 1211 Geneva 4, Switzerland; ^2^Department of Obstetrics and Gynaecology, Faculty of Medicine, University of Geneva, 30 bd de la Cluse, 1211 Geneva 4, Switzerland

## Abstract

Glucose-regulated protein of 78 kD (GRP78) is a chaperone protein mainly located in the endoplasmic reticulum (ER). This protein is normally present at low levels in adult cells but its expression is triggered by ER stress including glucose deprivation and hypoxia. In tumor cells, it is overexpressed with fraction of protein found at the cell surface. This paper presents the physiology of GRP78 in the context of ovarian cancer and its potential use as drug delivery systems targeting ovarian cancer cell.

## 1. Introduction

Glucose-regulated protein 78 (GRP78) is an endoplasmic reticulum (ER) chaperone protein belonging to the heat shock protein 70 family. It consists of two functional domains, a 44 kDa N-terminal ATPase and a 20 kDa C-terminal polypeptide-binding domain, and a variable 10 kDa C-terminal tail of unknown function.

This protein, as other members of this family, plays an essential role in protein biosynthesis (for review, see [1]). It facilitates folding and assembly of newly synthesized proteins and prevents intra- or intermolecular aggregation during stress conditions [2, 3]. GRP78 expression is induced by a variety of environmental and physiological stress conditions leading to impairment of essential ER functions and homeostasis in order to protect organs and tissues against apoptosis [4]. Its expression also varies with developmental stages and tissue specificity. A low basal level is identified in most adult tissues whereas it is highly induced in cancer [5, 6]. GRP78 expression is induced under such conditions as hypoxia and nutrient deprivation, partially explaining its high level in tumour cells [7].

GRP78 generally resides inside the ER lumen. However, GRP78 is also found at the cell surface in a wide variety of cancer cells, including neuroblastoma, lung adenocarcinoma, colon adenocarcinoma, ovarian tumour cells [8], prostate cancer [9], proliferating endothelial cells, and, more generally, stressed tumour cells [10]. It is still unknown how GRP78 localizes to the various cellular compartments, and its physiological role at the cell surface membrane is still not fully understood. A hypothesis is that upon GRP78 overexpression, it escapes to ER retention and reaches cell surface. Some proteins are involved in GRP78 relocation, as MTJ-1 and Par-4 [11, 12]. Through its binding to other proteins at the cell surface, GRP78 mediates cell-signalling pathways. For example, cell surface GRP78 acts as a receptor for alpha-2-macroglobulin, leading to activation of PAK-2, to induction of cell motility [12, 13], and to activation of MAPK and PI3K pathways which promote proliferation and survival in a variety of tumours [14, 15]. Other proteins have been identified as partners of cell surface GRP78 such as Cripto I [16], angiogenesis inhibitor plasminogen kringle 5 [17], Par-4 [18], or MHC-I molecule [19].

## 2. GRP78 and Its Role in Cancer

In a variety of cancer cells and solid tumours (breast, lung, prostate and ovarian cancers, melanoma, and glioma cells), the level of GRP78 expression is highly induced and could be essential for the survival of stressed cells such as cancer cells. Its expression correlates with malignancy, metastasis development, and drug resistance [9, 20–24]. It was shown that knockdown of GRP78 inhibits tumour cell invasion *in vitro* as well as tumour growth and metastasis aggressiveness in xenograft models [25, 26], suggesting an important role of GRP78 in cancer progression. However, the mechanism whereby GRP78 promotes growth and metastasis is just emerging. The presence of GRP78 at the cell surface of highly metastatic cancer cells tends to suggest that it might mediate signal transduction pathways inducing proliferation and invasion [14].

In xenograft models treated with antivascular and antiangiogenic agents, GRP78 induction is most important in tumour cells bordering necrotic regions induced by the treatment [5]. Chemoresistance of various cancer cells correlates with GRP78 expression and apoptosis inhibition [26–28]. This could be due to the fact that GRP78 can interact and inhibit the activation of apoptosis pathway components as described with caspase-7 [10] or p53 [29]. It can also bind to and inhibit the activation of BIK, BAX, and prevent cytochrome c release from mitochondria [30–32]. Furthermore, GRP78 forms a complex with other proteins and may indirectly decrease the activity of proapoptotic components.

It was recently found that GRP78 could play another important role in cancer progression in regulating VEGF-induced endothelial cell proliferation through the VEGF-MAPK signal cascade [33].

## 3. GRP78 Autoantibodies

GRP78 is overexpressed and relocated at cell surface of various cancer cells. It represents a potent biomarker of cell invasion, but its level may be too low to be detected in serum of women diagnosed with cancer. Mintz et al. have demonstrated the presence of GRP78 autoantibodies in patients with prostate cancer and suggested that GRP78 could act as a target of antibodies in these patients [9]. A strong and specific positive correlation was observed between serum reactivity to GRP78, development of metastatic androgen-independent disease, and shorter overall survival. Moreover, these antibodies do not seem to be increased in serum of patients with lung, breast, and ovarian cancer compared to control, suggesting specificity towards prostate cancer [9]. However, GRP78 autoantibodies were also identified in sera of mice bearing lung tumour as a model, and the titer was associated with the detection of primary tumour and metastases earlier than clinical identification. These observations suggest their potential utility in cancer detection and prognosis [34]. GRP78 autoantibodies were detected in serum of patients with gastric cancer, melanoma, and ovarian cancer but it is not clear if their level increases with stage of disease [35–38].

Circulating autoantibodies against GRP78 purified from prostate cancer patients were able to bind to GRP78 expressed at the surface of tumour cells, to the same site as the one recognized by its physiological agonist, the alpha2-macroglobulin [39]. This binding promoted proliferation of prostate cancer lines. Moreover, these antibodies protected cells from apoptosis induced by tumour necrosis factor alpha [39], suggesting that they could facilitate the emergence of more aggressive prostate cancer cell phenotype. In contrast, commercial antibodies against the C-terminus of GRP78 could act as antagonists and inhibit cellular proliferation and promote apoptosis [15, 40]. This contradicting observation suggests that the role of anti-GRP78 antibodies in tumour development may be dependent on the nature of the GRP78 epitope recognized by the antibodies. Autoantibodies against GRP78 isolated from serum of melanoma patients were also found to promote tumour growth [41]. This activity could depend on glycosylation of antibodies [36].

## 4. GRP78 in Ovarian Cancer

Epithelial ovarian cancer (EOC) accounts for the vast majority of ovarian malignancies. EOC survival rate is dependent on disease stage at the time of diagnosis. When diagnosed at stage III or more advanced stage, the 5-year mortality rate is close to 70% [42]. Due to the lack of specific symptoms and reliable ovarian cancer biomarkers, 75% of EOC patients have advanced stage disease at presentation, making EOC the most lethal gynecologic malignancy. The excellent survival rates for women with early stage disease provide a strong rationale to support research effort in developing strategies to identify the disease before it spreads outside the pelvis. Currently, it remains a big challenge.

Recently, researches have moved away from cytotoxic drugs to new targeted therapies, such as vascular endothelial growth factor (VEGF) inhibition and other agents that inhibit angiogenesis or cell-signaling pathways. Targeted therapy is now coming to the forefront of research and clinical trials in order to overcome resistance to cytotoxic drugs. The most promising at this time are angiogenesis inhibitors. Deeper understanding of cell-signaling pathways in ovarian cancer is needed to develop innovative strategies to improve outcome of EOC patients.

Despite the great interest of GRP78 in cancer development and progression, few data is available on GRP78 and ovarian cancer. First evidence of GRP78 as antigen associated with ovarian cancer was brought in 1997 by the detection of humoral immune response to GRP78 in ovarian cancer patients [43]. This presence is certainly associated to the expression of membrane GRP78 in ovarian cancer cells [8, 44]. Sera from ovarian cancer patients failed to recognize GRP78 on normal ovarian tissue suggesting that this antigen is unique to cancer [43]. However, Mintz et al. described the presence of GRP78 autoantibodies in serum of control female and the lack of difference between the level in ovarian cancer and control patients [9]. The same observation was recently reported by Lu et al. [44]. The level of GRP78 autoantibodies remains controversial since it was recently suggested that GRP78 autoantibodies increased with ovarian cancer stage [37] whereas Cohen and Petignat described the opposite [35]. There might be a variety of reasons why results are different, such as the methods of GRP78 autoantibodies detection (ELISA or immunoblot) or different sample size.

If some authors focused on GRP78 autoantibodies as prognostic marker of ovarian cancer, none reported a possible correlation between GRP78 level in ovarian cancer tissue and disease stage and chemotherapy resistance as suggested in lung and breast cancer, melanoma, and glioma cells [27, 28, 45–47].

## 5. GRP78 as a Recognition Element for Drug Targeting in Ovarian Cancer

Targeted therapy consists in the design and application of drugs specifically directed against well-defined targets that are critical for tumor survival and not compromising for normal organs and tissues. It may also involve the recognition of a molecular entity specific to the organ or cells of interest. This strategy takes all its sense when cancer mass is spread as micrometastasis as encountered in ovarian cancer. Specific localisation to the cancer cells will moreover provide safer therapy by reducing the high toxicity of chemotherapeutic drugs and the adverse effects related to the unfavourable biodistribution to both cancerous and healthy tissues. Targeted therapy is usually mediated via a ligand specific to a molecular target overexpressed or ideally exclusively expressed at the cells of interest.

GRP78 represents a very interesting target to be associated with drug delivery systems, as it is specifically expressed at cancer cell membranes.

Arap et al. designed two ligand peptides to specifically bind to the GRP78 expressed at cell surface [48]. After intravenous administration, these peptides were shown to be able to target prostate or breast cancer cells implanted in mice models whereas they were not detected in healthy tissues. The ligand peptides were linked to a proapoptotic peptide before systemic administration to tumour-bearing mice weekly for 4 weeks. Tumour volume was significantly smaller in chimera-treated groups compared to animals receiving either the vehicle or unconjugated mixture of the ligand peptides with the proapoptotic peptide.

In another study, GRP78 was identified as the receptor for the best candidate of a cohort of cyclic peptides screened for internalisation in melanoma cell lines, the “Pep42” [49]. After combination with quantum dots, cellular uptake and ER localisation of the conjugate were observed *in vitro*. Furthermore, direct combination of the cyclic peptide with taxol leads to an increased apoptosis rate *in vitro* in melanoma cells compared to free taxol. A control construct where taxol was conjugated to a linear peptide analogue had a weaker effect compared to taxol alone. The ability of Pep42 to selectively bind to human cancer cells was further tested with melanoma cells (Me6652/4), lung adenocarcinoma cells (A549), osteosarcoma cells (SJSA-1), hepatoma cells (HepG2), and two normal fibroblast cell lines [50]. The peptide bound to cancerous cells whereas only limited recognition was observed with normal cells. Selective apoptosis was induced in cancer cells and not in normal cells when Pep42 was linked to a proapoptotic peptide. Pep42 conjugated with quantum dots were administered to tumour-bearing animals. The conjugates concentrated in the tumour tissue without accumulation in other organs, demonstrating the specificity of the targeting.

These data support that cancer cells expressing the GRP78 at their surface are more sensitive to cytotoxic drugs when these are conjugated to recognition elements targeting the GRP78. Direct ligation of a ligand to a drug molecule is a common mean to achieve targeting. However, it needs chemical modification of the active compound and the further release of the drug that may compromise the pharmacological efficiency. Therefore, other approaches involving synthetic drug carriers have been developed. The drug is encapsulated or associated with a polymer or lipid core that is decorated at its surface with a recognition moiety.

Pegylated liposomes were surface-modified by Katanasaka et al. [33] with the peptide developed by Arap et al. [48]. It was shown *in vitro* that the liposomes were able to target VEGF-activated HUVEC cells as well as DU145, a prostate cancer cell line [48]. Biodistribution studies in mice demonstrated a preferable localisation of the targeted liposomes to the vasculature and the tumour tissue with no accumulation in normal tissues except for the spleen. Furthermore, the targeted liposomes were loaded with doxorubicin. In the dorsal air pouch model in mice implanted with C26 tumours, these liposomes suppressed the tumour-induced vascularisation angiogenesis whereas untargeted liposomes were not as efficient. Survival rate was also significantly increased in mice treated with targeted liposomes compared to sucrose solution or untargeted liposomes. This study shows the interest of using GRP78 as a target element to bring large amount of drug to a tumour via colloidal carriers. It also shows that GRP78 can be a target for cancer antineovascularisation therapy.

A more recent study reports the development of paclitaxel-loaded polyester nanoparticles conjugated with a GRP78-recognising peptide [51]. This approach proved to increase paclitaxel concentration and apoptosis in irradiated breast carcinoma in mice for up to 3 weeks. No significant tumour growth delay was observed when free paclitaxel or untargeted nanoparticles were used after irradiation compared to irradiation alone.

These studies strengthen the significance of using GRP78 as a targeting moiety for the development of more efficient anticancer treatments. So far, to our knowledge, only peptides have been used as recognising entity, and no studies on ovarian cancer has been reported. Antibodies against GRP78 may be used for their targeting capacities; however, they can also be of advantage as antitumoral agent as was demonstrated by Cohen and Petignat [35]. As proof of principle, we recently combined paclitaxel-loaded nanoparticles with anti-KDEL (NPs-Tx-KDEL) antibodies (unpublished data). Briefly, the results showed that antibody-coated particles presented a higher binding to the cells even though the internalization rate appeared limited. Moreover, despite the fact that KDEL antibodies exhibit unforeseen antiapoptotic properties (in the opposite to purified GRP78 autoantibodies), NPs-Tx-KDEL significantly increased sensitivity of Bg-1, an ovarian cancer cell line, to the drug compared to other treatments (free paclitaxel, unloaded carrier, or untargeted nanoparticles).

In summary, GRP78 appears of great interest as prognostic marker and therapeutic target for various types of cancer. Exciting data have been gathered regarding its role in the development of cancer, and a few studies have shown interesting prospective in using this antigen as a receptor for targeted therapy. Nevertheless, very few studies investigated GRP78 and anti-GRP78 autoantibodies in ovarian cancer. If potentiality for this protein has been suggested, no data is so far available to show its interest as marker or therapeutic target of ovarian cancer. Development of targeted drug delivery systems offers the possibility to deliver a high concentration of chemotherapeutic agents to the right place. Furthermore, if chosen wisely, the antibodies against the GRP78 may have anticancer activity by themselves.
